# Immobilization of Hyperthermostable Carboxylesterase EstD9 from *Anoxybacillus geothermalis* D9 onto Polymer Material and Its Physicochemical Properties

**DOI:** 10.3390/polym15061361

**Published:** 2023-03-09

**Authors:** Ummie Umaiera Mohd. Johan, Raja Noor Zaliha Raja Abd. Rahman, Nor Hafizah Ahmad Kamarudin, Wahhida Latip, Mohd Shukuri Mohamad Ali

**Affiliations:** 1 Enzyme and Microbial Technology Research Centre, Faculty of Biotechnology and Biomolecular Sciences, Universiti Putra Malaysia, Serdang 43400, Selangor, Malaysia; 2 Department of Biochemistry, Faculty of Biotechnology and Biomolecular Sciences, Universiti Putra Malaysia, Serdang 43400, Selangor, Malaysia; 3 Department of Microbiology, Faculty of Biotechnology and Biomolecular Sciences, Universiti Putra Malaysia, Serdang 43400, Selangor, Malaysia; 4 Centre of Foundation Studies for Agricultural Science, Universiti Putra Malaysia, Serdang 43400, Selangor, Malaysia

**Keywords:** carboxylesterase, polymer, immobilization, thermostable, stability

## Abstract

Carboxylesterase has much to offer in the context of environmentally friendly and sustainable alternatives. However, due to the unstable properties of the enzyme in its free state, its application is severely limited. The present study aimed to immobilize hyperthermostable carboxylesterase from *Anoxybacillus geothermalis* D9 with improved stability and reusability. In this study, Seplite LX120 was chosen as the matrix for immobilizing EstD9 by adsorption. Fourier-transform infrared (FT-IR) spectroscopy verified the binding of EstD9 to the support. According to SEM imaging, the support surface was densely covered with the enzyme, indicating successful enzyme immobilization. BET analysis of the adsorption isotherm revealed reduction of the total surface area and pore volume of the Seplite LX120 after immobilization. The immobilized EstD9 showed broad thermal stability (10–100 °C) and pH tolerance (pH 6–9), with optimal temperature and pH of 80 °C and pH 7, respectively. Additionally, the immobilized EstD9 demonstrated improved stability towards a variety of 25% (*v*/*v*) organic solvents, with acetonitrile exhibiting the highest relative activity (281.04%). The bound enzyme exhibited better storage stability than the free enzyme, with more than 70% of residual activity being maintained over 11 weeks. Through immobilization, EstD9 can be reused for up to seven cycles. This study demonstrates the improvement of the operational stability and properties of the immobilized enzyme for better practical applications.

## 1. Introduction

For several decades, efforts have been made to replace chemical catalysts with enzymes for multidisciplinary applications. In comparison to the conventional organic syntheses, biocatalytic syntheses typically take less time, use less energy, and produce less waste. Enzymes participate in speeding up chemical reactions in mild environments with very high substrate specificity. Among the great variety of enzymes, carboxylesterase has become the enzyme of choice due to its ability to efficiently catalyze many chemical reactions, including transesterification, hydrolysis, esterification and alcoholysis [1,2,3]. It is a cofactor-independent enzyme and has high regio- and stereoselectivity. Carboxylesterases are prevalent in nature [4]. Of all sources, bacterial carboxylesterase is the main biocatalyst used in practical applications, such as in the production of detergent, textile, paper and the synthesis of optically purified compounds [5,6,7,8]. Despite this versatility, the applied free enzyme generally displays poor stability against high temperature, pH, and some chemical reactions, such that environmental circumstances can alter its conformation, leading to either reversible or irreversible deactivation. Additionally, the lack of a recovery process and long-term stability of the free enzyme often presents challenges for its further application [9,10,11]. To counteract these inherent drawbacks, the employment of enzyme immobilization has been recognized to be the ideal alternative.

During the immobilization process, the enzyme is chemically or physically incorporated into a selected solid support [12]. Immobilization can enhance enzyme stability against denaturation caused by heat, high pH, high ionic strength, chemical contaminants, or proteolysis. In fact, the homogeneity of the immobilization system allows easy recovery of the enzyme and product [13,14]. Through this technique, the enzyme is easy to control and separate from a reaction mixture to avoid contamination [15,16]. The prime requirement for satisfying industrial demand is the repeated use of a catalyst. An immobilized enzyme can be used repeatedly and continuously. In this way, the enzyme can be utilized for a long time before it needs to being replaced with a new enzyme, which greatly reduces enzyme cost and improves the viability of biotechnological processes [17,18]. Many areas, such as food processing, oleochemical industries, biofuel production, and bioremediation, will benefit from the outstanding properties of immobilized enzymes [19,20,21,22]. It is important to note that an inappropriate support used in enzyme immobilization may induce conformational modifications that can lead to partial activity loss. Therefore, the selection of the support is a very important step.

Previously, carboxylesterase has been immobilized onto several supports, including polypropylene, magnetic nanoparticles and silica particles [1,23,24]. To the best of our knowledge, the immobilization of carboxylesterase on polystyrene-divinylbenzene (PS-DVB) copolymer has not yet been reported in the literature. Polystyrene divinylbenzene copolymer, polyacrylamides and polyamides are the most commonly used hydrophobic supports for biocatalysts [25]. Polystyrene-divinylbenzene copolymer is economical and widely available in the market [26,27]. Considering the large available surface area, appropriate pore size, non-toxicity and dispersible properties, polystyrene-divinylbenzene copolymer is the ideal support for enzyme immobilization.

In our previous study, carboxylesterase EstD9 from *Anoxybacillus geothermalis* D9 was characterized and identified as a hyperthermostable enzyme [28]. Although carboxylesterase EstD9 has high potential as a biocatalyst, the enzyme in its free state can be disadvantageous for industrial purposes and its longevity has not been studied. In this work, the immobilization of carboxylesterase onto Seplite LX120 using adsorption method was investigated for the first time. The immobilization time and enzyme loading were optimized to obtain the maximum adsorption yield. Immobilization efficacy was confirmed by physicochemical characterization using several analytical methods, including SEM, FT-IR and BET. Additionally, biochemical characterization, reusability and storage stability of immobilized EstD9 at different temperatures were also investigated.

## 2. Materials and Methods

### 2.1. Materials

*Escherichia coli* BL21 (DE3) harbouring a pET51b(+)/*estD9* expression vector was obtained from the Enzyme and Microbial Technology Research Centre (EMTech) laboratory. The strain was stored in 20% glycerol stock at −80 °C. Seplite LX120 (Series number: Q/LX078-2017) was purchased from Sunresin New Materials Co., Ltd. (Shaanxi, China) in the form of white opaque beads. The average particle size and density were 0.125–0.30 mm and 1.05–1.15 g/mL, respectively.

### 2.2. Enzyme Preparation

The carboxylesterase gene from *Anoxybacillus geothermalis* D9, abbreviated as *estD9*, was used in this study. The expression of recombinant EstD9 and purification by single-step affinity chromatography were performed in accordance with the procedures described by Johan et al. [28]. Enzyme activity and protein content were measured for future experiments.

### 2.3. Protein Content Determination

The Bradford method was employed with slight modifications to quantify the protein concentration [29]. In a 96-well microplate, 200 µL of Bradford reagent was added to 10 µL of 5 mM NaCl and 10 µL of enzyme. The reaction mixture was incubated at room temperature for 2 min prior to reading of the absorbance at the 595 nm wavelength. The reaction mixture without the enzyme acted as a blank. Protein concentration was measured based on the bovine serum albumin (BSA) standard curve.

### 2.4. Immobilization of EstD9

The purified EstD9 was immobilized onto Seplite LX120 through an adsorption method. In order to obtain high immobilization yield, the enzyme was subjected to two variables, which were adsorption time and enzyme loading. The optimization of immobilization was carried out using one-variable at a time approach. The attachment of carboxylesterase to the support was determined by measuring the protein content, and the activity was calculated under standard assay conditions.

#### 2.4.1. Effect of Adsorption Time

**Figure 1:**
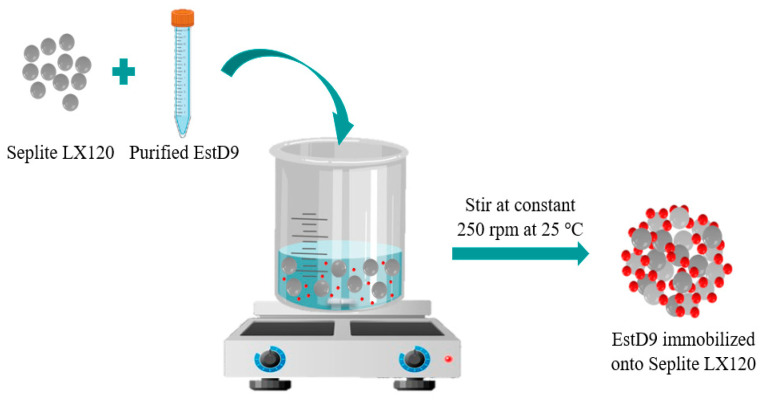
Schematic illustration of enzyme incorporation onto polymer support by physical adsorption.

Figure 1 illustrated the adsorption method employed for EstD9 immobilization. The enzyme solution was added into a 50 mL conical flask with 5 mg of protein per g of Seplite LX120 in a buffer system of 50 mM of Tris-HCl (pH 8). The effect of immobilization time was determined by stirring the purified enzyme with the support Seplite LX120 at 0 min, 15 min, 30 min, 45 min, 60 min, 75 min and 90 min at a constant stirring rate of 250 rpm at room temperature. Afterwards, the unbound enzyme was filtered out of the mixture and the supernatant was collected for protein content measurement. The support material was repeatedly washed with a buffer to remove the eluate. Following that, the bound support was dried in a fluid bed dryer and enzymatic activity was measured. The initial protein content of the sample at 0 min and the final concentration were taken for the quantification of immobilization yield using Equation (1).

(1) $$\mathrm{Immobilization}\;\mathrm{yield}\;(\%)=\frac{C_{0}-C_{1}}{C_{0}}\times 100$$

*C*_0_: initial protein concentration; *C*_1_: final protein concentration (mg/mL).

#### 2.4.2. Effect of Enzyme Loading

Enzyme loading was varied with protein concentrations of 0.25 mg/mL, 0.5 mg/mL, 0.75 mg/mL, 1.0 mg/mL, 1.25 mg/mL and 1.5 mg/mL per g of support. Approximately 10 mL of enzyme solution was added to 1 g of Seplite LX120 and stirred for 1 h (based on the optimum immobilization time). The supernatant was collected to measure the residual protein content. The adsorption yield was calculated by comparing the initial protein concentration to the unbound enzyme protein concentration. The immobilized enzyme was assayed based on the standard assay condition.

### 2.5. Enzyme Assay

A modified *p*NP assay was employed to measure the hydrolytic activity of immobilized EstD9. A mixture of 220 µL of 20 mM of sodium phosphate buffer (pH 7.4), 20 µL of 5 mM *p*-nitrophenyl butyrate (C4), and 10 mg of immobilized enzyme was incubated at 80 °C with shaking at 150 rpm for 10 min. The assay was done in triplicate and the control reaction mixture without the enzyme was regarded as the control. Absorbance was measured at the 410 nm wavelength using a UV spectrophotometer (Bio-Rad, Hercules, CA, USA). The activity of the immobilized enzyme was expressed in unit/gram (U/g). A unit of immobilized enzyme activity was defined as the amount of enzyme required to release one µmol of *p*-nitrophenol per min under standard assay conditions. The hydrolytic activity of the immobilized enzyme was measured using Formula (2):

(2) $$\mathrm{Carboxylesterase}\;\mathrm{activity}\;(U/g)=\frac{\Delta_{410}\;}{m\times t\times w}$$

Δ_410_: Sample absorbance at 410 nm subtracted from control absorbance; *m*: gradient of *p*NP standard curve; *t*: reaction time; *w*: weight of support in grams.

### 2.6. Structural and Morphological Characterization

The morphology and surface of the bound and unbound Seplite LX120 were determined by scanning electron microscopy using a LEO 1455 VPSEM (Netherlands) with EDX attached operating at 10 kV. The samples were mounted on specimen stubs and coated with a gold layer. Direct observations were performed at magnifications of 20×, 5000× and 10,000×.

Fourier-transform infrared (FT-IR) spectroscopy analysis was performed on the immobilized enzyme, free enzyme, and unbound support to analyze the binding of the enzyme onto the carrier using a Thermo Nicolet 6700 FT-IR spectrometer (Thermo Scientific, Waltham, MA, USA). The bound and unbound supports were ground to a powdery form and pressed into a thin KBr pellet prior to analysis. The structural analysis was performed in a frequency range of 450 to 4000 cm^−1^. Spectra were obtained at a spectral resolution of 4 cm^−1^ with an average of 32 scans.

The specific surface area and pore structure were analyzed by nitrogen adsorption-desorption at 150 °C with a 10 s equilibration time. The samples were degassed for 2 h at 300 °C using vacuum prior to adsorption. The Brunauer–Emmett–Teller (BET) method was used to calculate the specific surface area, and the total pore volume was measured using the Barrett–Joyner–Halender (BJH) method.

### 2.7. Biochemical Characterization

#### 2.7.1. Effect of Temperature on Activity and Stability

The optimum temperature for bound *A. geothermalis* D9 carboxylesterase was investigated by measuring the enzymatic activity of 10 mg of the immobilized enzyme under standard assay procedures at different temperatures, ranging from 10–100 °C, in 20 mM of sodium phosphate buffer, pH 7.4 for 10 min. For temperature stability, the immobilized EstD9 was pre-incubated for 30 min at a set temperature range, from 10 to 100 °C, prior to enzymatic assay. The residual activity was measured and expressed as the percentage of relative activity.

#### 2.7.2. Effect of pH on Activity and Stability

The optimal pH of immobilized EstD9 was determined by performing assays in buffer systems with different pH values (pH 4–11). The buffers used were sodium acetate (pH 4–5), sodium phosphate (pH 5–7), Tris HCl (pH 7–9), and glycine-NaOH (pH 9–11). The pH stability for the immobilized EstD9 was tested by performing an enzymatic assay of the remaining activity after 30 min of pre-incubation in different buffer systems. The relative activity of immobilized enzyme was calculated with respect to the highest activity (100%).

#### 2.7.3. Effect of Organic Solvents on Activity

The effect of organic solvents was tested with the addition of various 25% (*v*/*v*) organic solvents, including DMSO, acetonitrile, 1-propanol, 2-propanol, butanol, benzene, toluene, heptanol, octanol and xylene. The immobilized enzyme was pre-incubated with the organic solvents in 20 mM of sodium phosphate buffer, pH 7.4 for 30 min at 70 °C. Afterwards, enzyme assay was performed under standard condition. The percentage of relative activity was measured according to the activity of the solvent-free reaction (100%).

#### 2.7.4. Effect of Metal Ions on Activity

The effect of various metal ions on immobilized EstD9 was determined by pre-incubating the enzyme with final concentrations of 1 mM and 5 mM of Na^+^, K^+^, Mg^2+^, Ca^2+^, Mn^2+^, Cu^2+^, Zn^2+^ and Fe^3+^ at 70 °C for 30 min and performing enzyme assay. The relative activity was measured by comparing the enzyme activity with the reaction without additive (100%).

### 2.8. Storage Stability

The bound and the free enzymes were stored at 4 °C and 25 °C. The samples were stored for 11 weeks and taken out periodically every 7 days for residual activity determination. The storage at week 0 served as the control (100%).

### 2.9. Reusability

The reusability of the immobilized enzyme was performed under standard assay conditions with enzyme loading of 10 mg, 20 µL of 5 mM *p*-nitrophenyl butyrate, and 20 mM of sodium phosphate buffer (pH 7.4) at 80 °C for 10 min. Then the immobilized enzyme was washed for several times with a buffer and dried using a fluid bed dryer prior to subsequent reaction cycles. The experiments were performed eight times under the same conditions. The activity and conversion measurements were calculated based on the first trial reaction at cycle 0 (regarded as 100%).

### 2.10. Statistical Analysis

All experiments were carried out in triplicate, and the results were calculated using Microsoft Excel spreadsheets to produce the mean value and the standard deviation reported for each experiment.

## 3. Results and Discussion

### 3.1. Immobilization of Purified EstD9

**Figure 2:**
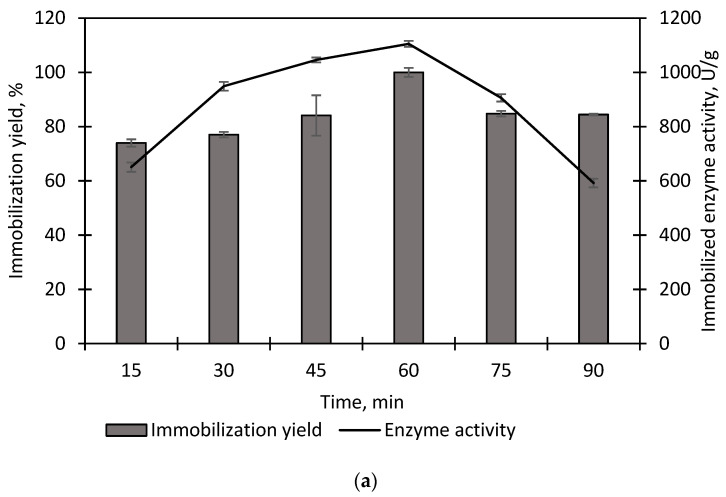

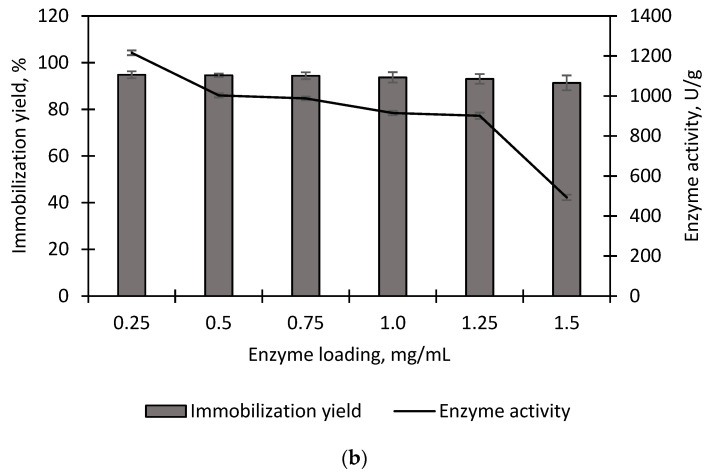
(**a**) Time course for EstD9 adsorption profile onto Seplite LX120; (**b**) effect of enzyme loading during immobilization procedure.

In this study, direct physical adsorption was employed with interacting forces of ionic interaction. An advantage of physical adsorption compared to other immobilization methods is that the enzyme does not have to be highly pure [30]. Therefore, the purification of recombinant EstD9 via one-step affinity chromatography was sufficient for the immobilization procedure. Various conditions for immobilization were investigated, with the aim of maximizing the yield. The reaction time for immobilization is important, as the denaturation of enzyme may occur during the process, thereby leading to enzyme deactivation [31]. A series of immobilization tests was performed on a varied time course (15 min to 90 min) in pH 8 at room temperature. The initial concentration of purified enzyme was kept constant at 0.5 mg/mL. As shown in Figure 2a, the highest immobilization yield was achieved during the first hour of reaction (100% immobilization yield, 1105.13 U/g). The initial increase in immobilization yield was due to the high availability surface area for enzyme attachment. However, the immobilization yield decreased to 84.8% and 84.46% at 75 and 90 min, respectively. The result indicated that a short time was sufficient for the purified enzyme to completely bind to the support, and that extending the time did not result in maximum immobilization yield. Prolonged time of reaction may cause enzyme leaching or detaching from the support due to a mechanical force effect. Therefore, rapid immobilization of EstD9 was needed in order to reduce the chance of enzyme leaching during the procedure. In contrast, the reported FAE from *H. insolens* took 26 h to achieve the maximum loading, and immobilization of the carboxylesterase gene (*est*_741_) from *Geobacillus uzenensis* required 14 h of reaction time [32,33].

The enzyme loading capacity was highly influenced by the specific surface area and the pore size of the support [34]. Seplite LX120 is a microporous support with a small pore size and large surface area for the attachment of an appropriate amount of enzyme onto its surface. Therefore, the immobilization of EstD9 was further optimized by varying concentrations of enzymes in a buffer system at pH 8 for 1 h at room temperature. As shown in Figure 2b, the support has a maximum adsorption capacity of 0.25 mg/mL with enzymatic activity of 1215.79 U/g. This result indicates that the enzymes were completely bound onto the surface of the Seplite LX120. However, increasing the enzyme concentration led to a reduction in adsorption capacity and enzyme activity. Immobilization yield decreased to 93.7% with 135.51 U/g activity when 1.0 mg/mL of enzyme was loaded. The onset of mass enzyme loading may cause the formation of multiple enzyme layers, preventing the excess enzyme from coming into contact with the external surface of the support. As a result, the support surface area reaches its saturation limit and the immobilization efficacy plateaued.

### 3.2. Morphological Characterization of Immobilized EstD9

**Figure 3:**
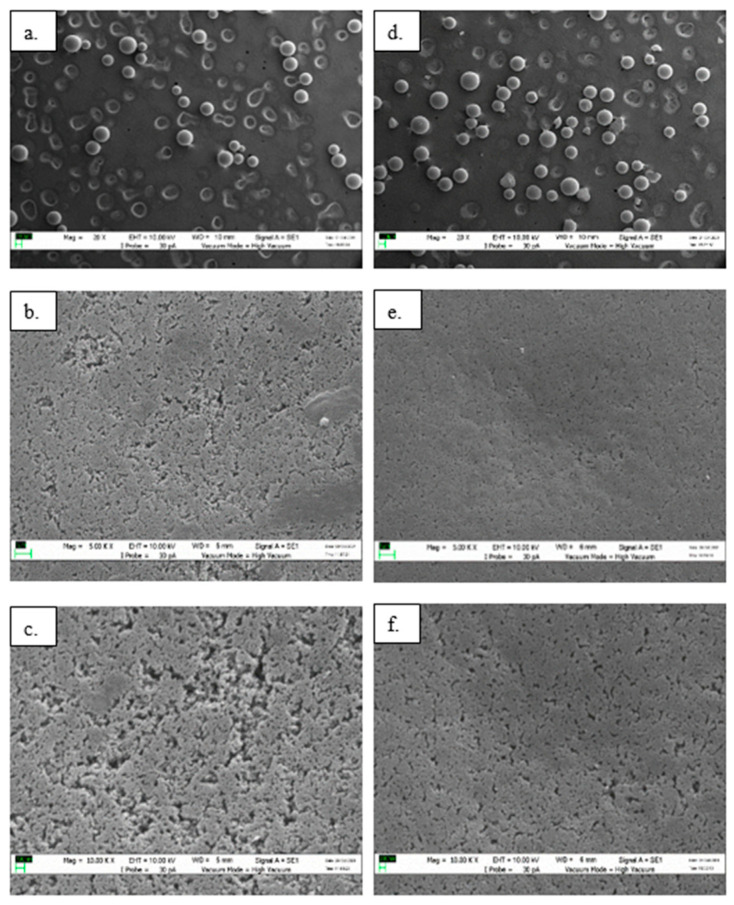
Surface morphology of Seplite LX120 before and after immobilization with EstD9. Images (**a**–**c**) show the empty Seplite LX120, while (**d**–**f**) show bound support of the enzyme at magnifications of 20×, 5000× and 10,000×, respectively.

Scanning electron microscopy (SEM) was used to analyze the surface characteristics, shape and size of the bound and unbound supports. At a magnification of 20×, the empty support materials were observed to have a regular rounded shape and smooth surface with a particle size distribution in the range of 125–300 µm, as shown in Figure 3a. Following the immobilization process, no significant differences in the dimensional and geometric shapes of Seplite LX120 were detected, except for some irregularly shaped beads due to mechanical force during stirring (Figure 3d). The support remained dispersed, similarly to the unbound support. This indicates that the immobilization process did not confer significant changes in particle size. The enlarged SEM images at magnifications of 5000× and 10,000× clearly showed that the unbound support had a microstructure with surface cracks (Figure 3b,c), whereas the bound support looked smoother with fewer cracks (Figure 3e,f). This suggests high coverage of the specific surface area of Seplite LX120 by the enzyme. The differences in the morphological surfaces of the immobilized enzyme, as shown by SEM images, served as an indication of successful enzyme immobilization. Similar morphological characteristics were also observed for esterase from *Geobacillus* sp. [35].

### 3.3. BET Analysis of Seplite LX120 and Immobilized EstD9

**Figure 4:**
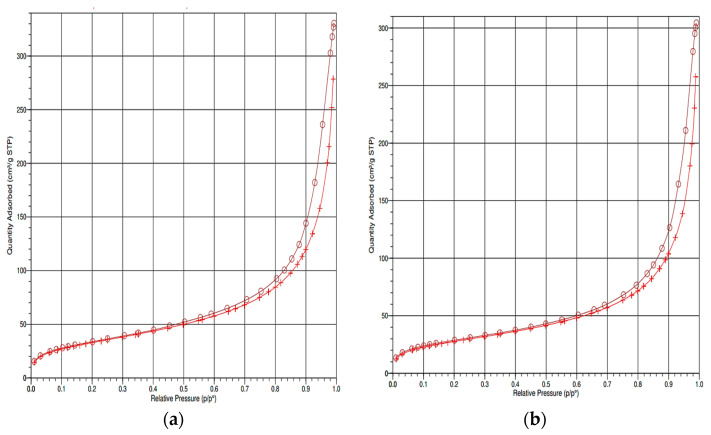
The N_2_ adsorption-desorption isotherm of BET analysis of Seplite LX120: (**a**) before immobilization; (**b**) after immobilization of EstD9. Desorption is denoted with symbol (ө), while adsorption is represented with symbol (+).

The porosity and surface area of the support were subjected to Brunauer, Emmett and Teller (BET) analysis. The total surface area was commonly determined using the BET method based on the relative pressure value (p/*p*^0^). In comparison to the unbound support, the immobilized support showed a decrease in specific surface area from 122.3 m^2^/g to 102.3 m^2^/g. Additionally, the total pore volume estimated from the amount of nitrogen adsorbed at p/*p*^0^ = 0.9 was decreased to 0.019 cm^3^/g after immobilization of EstD9. The reduction in specific surface area and pore volume of the Seplite LX120 suggested that the enzyme had effectively covered a significant amount of the surface area and pores. Figure 4 shows the nitrogen adsorption-desorption isotherms of empty and immobilized support feldspars at 77 K. The adsorption initially increases steadily at low pressure, followed by rapid uptake at saturated vapour pressure. According to the International Union of Pure and Applied Chemistry (IUPAC), both adsorption curves yield a reversible type II isotherm, which corresponds to non-porous or microporous materials [36]. This analysis clearly shows that nitrogen gas was adsorbed into the pores of the Seplite LX120 surface. The isotherm revealed that the empty support had a higher rate of nitrogen adsorption than the immobilized EstD9. This could be due to higher availability of the surface area compared to the bound support.

### 3.4. Structural Analysis of Immobilized EstD9

**Figure 5:**
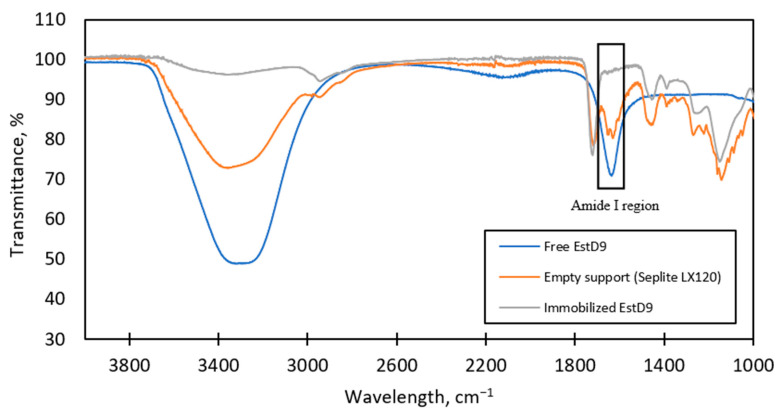
FT-IR spectral profile of free EstD9, empty Seplite LX120, and immobilized EstD9. Blue line represents free EstD9, while the empty Seplite LX120 and immobilized EstD9 are denoted in orange and grey lines, respectively.

To further confirm the binding of EstD9 onto the support, the free, unbound support and the immobilized support were analyzed by Fourier-transform IR (FT-IR) spectroscopy measurement. In this context, structural analysis of EstD9 when placed in contact with Seplite LX120 was performed on the basis of functional group formation. Peaks at certain wavenumbers correspond to specific chemical bonds. The spectral band in the wavelength range of 3000–3500 cm^−1^ was attributed to C-H and hydroxyl groups (O-H). The strongest peak in this region was observed for free EstD9, followed by Seplite LX120, due to the existence of water molecules (H-OH bending), which were not present in the bound Seplite LX120. After the incorporation of EstD9 onto the support, the absorption peak in this region was shifted to a lower frequency region, indicating the replacement of the OH group on the support by the enzyme (Figure 5). The band proximal to the 1600 to 1700 cm^−1^ range belonged to the functional region of amide I, which was associated with the C=O stretching and C-N groups [37]. This band is the most sensitive spectrum for protein structure determination associated with the overlapping of several structural elements such as α-helices, β-sheets, turns and random coils [38,39]. Strong absorption at 1600–1700 cm^−1^ was observed for the spectra of the free enzyme and the empty Seplite LX120, while the immobilized EstD9 showed no peak within that spectral range. This suggested that there were changes in the secondary protein structure of EstD9. In the case of the bound Seplite LX120, the weak spectral band in the amide I region formed due to the presence of primary amines on the external surface of the support. The result obtained was an indication that the immobilization was successful, owing to the interaction of the carbonyl group of the enzyme with the amino group of the support. In this sense, the presence of the active group facilitates the surface functionalization for enzyme attachment. Therefore, the attachment of the enzyme onto the support was confirmed based on the FT-IR pattern.

### 3.5. Effect of Temperature on Immobilized EstD9 Activity and Stability

**Figure 6:**
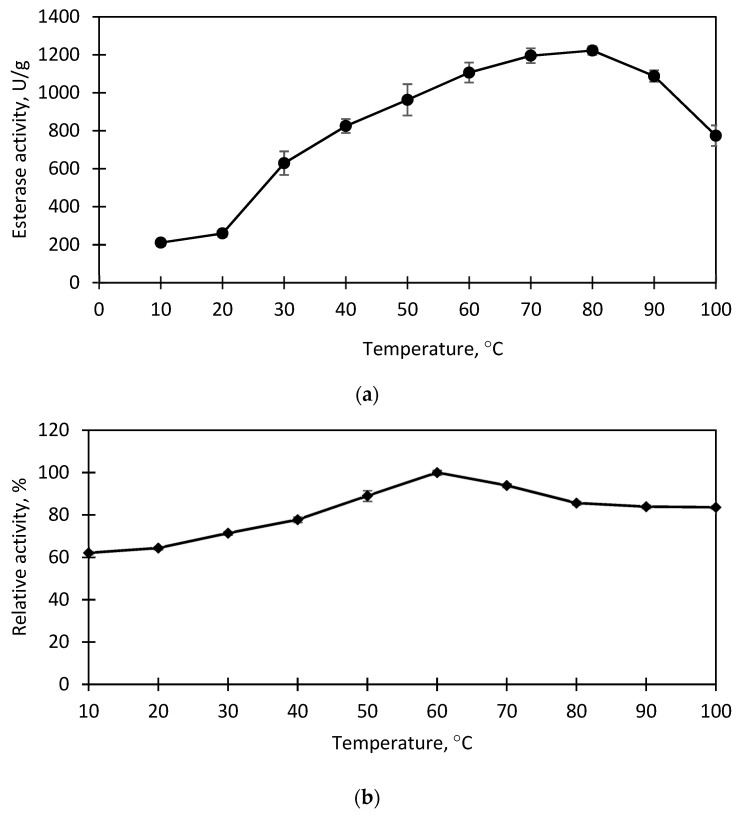
Effect of temperature on the immobilized EstD9: (**a**) activity; (**b**) stability. The immobilized EstD9 was treated in the temperature range of 10 to 100 °C. The maximal activity measured under standard condition is at the optimum temperature of the enzyme. The highest activity is regarded as 100% relative activity and the remaining activities are measured accordingly. Data represent the mean with ± standard deviation of three replicates (*n* = 3).

Biochemical studies primarily give an insight into enzyme nature and properties. Environmental factors, such as temperature, may influence enzymatic activity and stability. The immobilized EstD9 was treated at various temperature ranges from 10–100 °C for 10 min. As shown in Figure 6a, the optimal temperature of immobilized EstD9 appeared to be the same as that of the native enzyme at 80 °C [28]. The results indicated that the incorporation of the enzyme with the support material did not cause steric hindrance at high temperature, thereby allowing the preservation of the active site and making it accessible for substrate binding. Biocatalysts with high thermal stability are required in many modern biotechnological settings. To test its thermal stability, the immobilized enzyme was incubated at 10–100 °C for 30 min prior to enzyme assay. The enzyme retained more than 60% of its basal activity at all tested temperatures and reached its highest stability at 60 °C (Figure 6b). Similar to the corresponding free enzyme, the immobilized EstD9 had high heat sustainability in a wide temperature range [28]. This heat resistance could be attributed to the non-covalent multipoint attachment of the enzyme on the support, which reduced its conformational flexibility, making it more rigid [40]. Therefore, the movement restriction helps structure stabilization and activity retention. The result revealed that the immobilization process did not change the enzyme’s thermal stability. Such extreme-temperature enzyme stability could benefit various practical applications involving processing in harsh conditions.

### 3.6. Effect of pH on Activity and Stability of Immobilized EstD9

**Figure 7:**
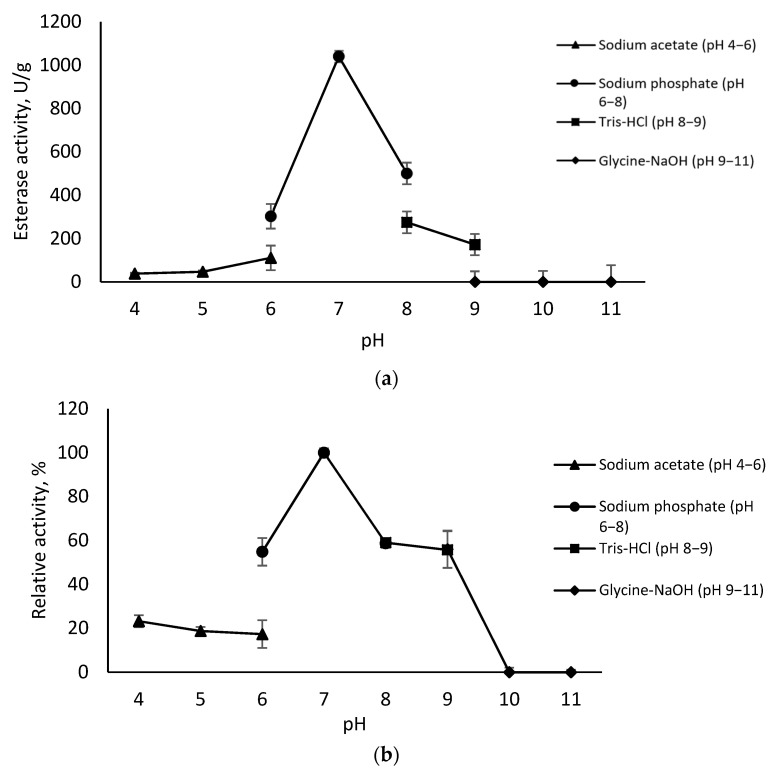
Effect of pH on the immobilized EstD9: (**a**) optimum; (**b**) stability. Enzymatic activity was measured at 80 °C in standard assay conditions. The relative activity at pH 8 was considered 100%. Error bars represent the standard deviation of means (*n* = 3).

Changes in pH condition can impact enzyme activity and stability. A slight fluctuation in pH may modify enzymatic activity. The pH profile of the immobilized EstD9 was investigated in different buffer systems from pH 4 to pH 11. As shown in Figure 7a, the activity and yield of the immobilized EstD9 was the highest at pH 7 in a 50 mM sodium phosphate buffer (1040 U/g), followed by pH 8 using 50 mM of Tris HCl (500 U/g). Our previous research on the behaviour of free EstD9 at different pH levels also showed maximal activity at neutral pH. Thus, the optimal pH for immobilized EstD9 was pH 7 and the environmental condition was similar to the free enzyme. These observations were similar to studies of immobilized EstSIT01 and HRP on carbon nanospheres [41,42].

Each immobilized enzyme displays a different ideal pH range to work efficiently. The stability of immobilized EstD9 was studied by pre-incubation at 70 °C for 30 min in buffers with different pH values (pH 4–11). EstD9 showed stability over a broad pH range (6–9), with more than 50% of relative activity retained (Figure 7b). The stability trend was consistent with immobilized titania-immobilized BCA-EctP1 [43]. Embedded in the solid surface, the enzyme was exposed to a different local microenvironment. The immobilized enzyme characteristics now depend on the nature of the support material. With Seplite LX120, the maximum stability shifts to neutral pH 7, differing from the free state (pH 8). Apparently, the enzyme exhibits higher stability at a higher pH, suggesting its preference for an alkaline environment. This can be explained by the possibility of the alteration of electron arrangement in the enzyme and the electric charge on the support. Immobilized EstD9 showed low enzymatic activity in extreme pH environments (pH 10–11). High acidity and alkalinity may disrupt the three-dimensional structure of protein, which prevents enzyme-substrate complex formation [44].

### 3.7. Effect of Organic Solvents on Activity of Immobilized EstD9

**Figure 8:**
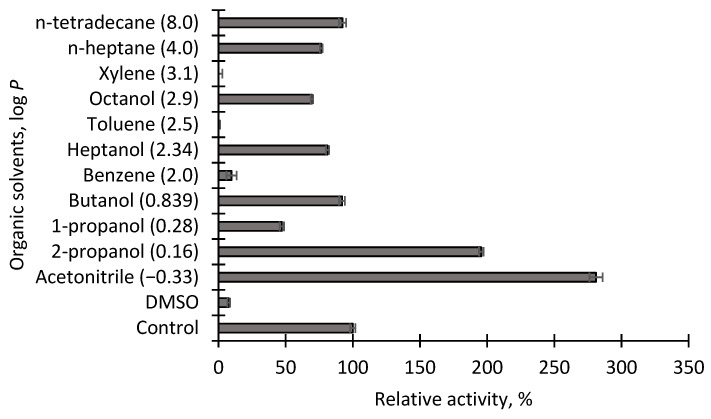
Effect of organic solvents on immobilized EstD9. The error bar indicates the standard deviation of triplicate assays (*n* = 3).

Organic solvent tolerance is another important parameter for evaluating the performance of an immobilized enzyme in industrial processing. Most synthetic reactions involve the usage of extremely harsh organic solvents. The degree of solvent tolerance is different for every individual enzyme. Therefore, the effect of various 25% (*v*/*v*) organic solvents on the stability of immobilized EstD9 was investigated. It was found that the solvent tolerance of EstD9 attached to a Seplite LX120 support differed from the free enzyme. In general, many carboxylesterases have been reported to be highly unstable in polar solvents, as they remove water solvation from the enzyme [45,46]. As observed in Figure 8, the immobilized EstD9 exhibited good stability to polar organic solvents (log *P* < 2.0), with the exception of DMSO and 1-propanol. The highest stability was achieved with acetonitrile, with which 281% of relative activities was retained in comparison to the untreated enzyme, followed by 2-propanol (195.6%). In other research, immobilized esterase from *Lysinibacillus fusiformis* AU01 maintained half of its relative activity in the presence of acetonitrile and 2-propanol [47]. The probable reason for the higher residual activity of the immobilized enzyme with acetonitrile compared to the corresponding free enzyme is the protection by the support material, thereby improving its structure sustainability against the solvent.

Most of the non-polar organic solvents (log *P* > 2.0) inhibited enzymatic activity of EstD9 from moderate to total loss of activity. However, 76.48% of the enzyme activity was retained with n-heptane and 92.32% of residual activity in the presence of n-tetradecane. Octanol and heptanol maintained more than half of relative activities with 69.6% and 81.2%, respectively. Meanwhile, no retention activity was observed when introduced with xylene and toluene. The probable reason is the interaction of the amino group on the support with the amino acid residues that are crucial for enzyme activity. Another probable factor that adversely affected catalytic function was conformational and substrate solubility modification by hydrophobic solvents [46].

### 3.8. Effect of Metal Ions on Activity of Immobilized EstD9

**Figure 9:**
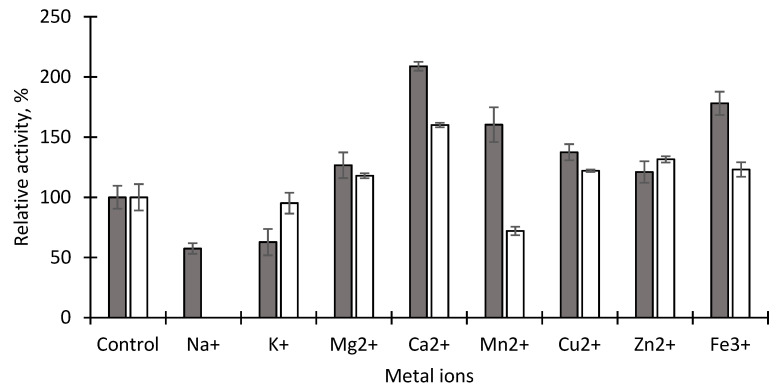
Effect of various metal ions on immobilized EstD9 activity. Relative activity was measured after pre-treatment with 1 mM (grey bars) and 5 mM of metal ions (white bars) for 30 min. The untreated sample was taken as the control with 100% relative activity. Error bars represent standard deviations (*n* = 3).

The effect of metal ions on immobilized enzymes can vary depending on the type of metal ion. Some metal ions can cause denaturation or aggregation of the enzyme, leading to decreased activity or even irreversible inactivation, while in other cases, they can affect the stability and longevity of immobilized enzymes. The enzymatic activities of immobilized EstD9 with metal ions at two different final concentrations (1 mM and 5 mM) were tested by incubating the treated enzyme at 70 °C for 30 min, followed by enzyme assay. As shown in Figure 9, the addition of 1 mM of Ca^2+^ stimulated immobilized EstD9 activity, with 208.84% of relative activity. The enzyme activity was also markedly enhanced to 117.88%, 160.36%, 137.44%, 121%, and 178.08% with the presence of 1 mM of Mg^2+^, Mn^2+^, Cu^2+^, Zn^2+^ and Fe^3+^, respectively. Higher concentrations of these metal ions did not confer any significant reduction in enzyme activity, with the exception of the heavy metal Mn^2+^. In comparison to 1 mM of Mn^2+^, a 5 mM concentration reduced the activity of immobilized carboxylesterase to 72.06% of relative activity. At a 1 mM concentration of group I metal ions (Na^+^ and K^+^), half of the enzyme activities were retained, with 57.44% and 62.76%, respectively. Increasing the concentration of Na^+^ to 5 mM, however, completely inhibited the enzyme activity. The probable reason was that the metal bound to the enzyme surface and interfered with its active site, leading to enzyme inactivation. In contrast, treatment with 5 mM of K^+^ did not alter the activity of immobilized EstD9.

In general, metal ions have the ability either to stabilize or disrupt the structure of proteins through an ion-mediated expansion of hydrogen bonding networks within the atoms. The Ca^2+^ activation of immobilized EstD9 may be caused by the stabilization of the enzyme in its active conformation, rather than being involved in a catalytic reaction. Positively charged Ca^2+^ ions can interact with negatively charged amino acid residues on the protein surface. This interaction can help to neutralize the negative charge on the protein surface and reduce repulsive electrostatic interactions between protein molecules, resulting in increased protein stability [48,49]. This finding was in contrast with that for the immobilized PEL3/Ca_3_(PO_4_)_2_ hybrid nanoparticles, in which enzyme activity remained only 13% in the presence of Ca^2+^ [50]. In general, increasing the concentration of metal ions had a moderate impact on the immobilized EstD9 activity.

### 3.9. Storage Stability and Reusability

**Figure 10:**
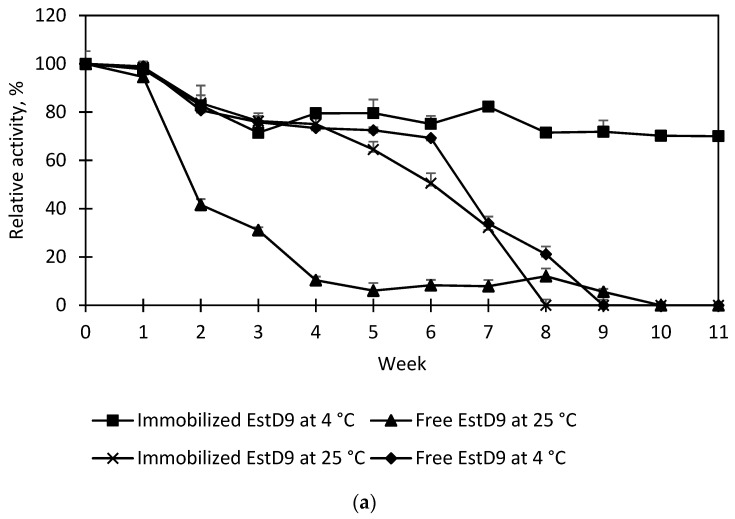

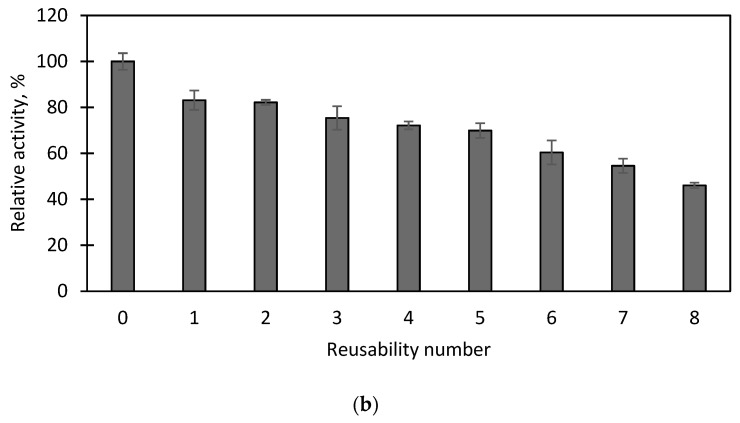
(**a**) Storage stability of free and immobilized EstD9; (**b**) reusability test of immobilized EstD9 for 8 assay cycles. The relative activity indicated the mean of standard deviation of triplicate assays (*n* = 3).

In practise, longevity takes precedence over the activity of an enzyme. Immobilization was observed to increase the stability of the enzymes in many cases [31,51]. A number of studies have demonstrated that enzymes are exceedingly stable in a dry state. The storage stability of EstD9 was evaluated over a period of 14 weeks at 25 °C and 4 °C. Residual activities were periodically measured every 30 days. As shown in Figure 10a, long-term storage of free EstD9 at 25 °C indeed impacted enzyme activity, such that a 90% of reduction in relative activity was observed. At 4 °C, the free enzyme showed a decline in residual activity during 7 weeks of storage and lost nearly all activity after 8 weeks. Under the same storage conditions, the immobilized EstD9 exhibited the lowest enzyme leaching, maintaining 70% of initial activity over 11 weeks. This result suggested that physical adsorption can prevent the dissociation of the enzyme from the support material, most likely depending on the surface charge of the support and the interaction with the opposite charge of the enzyme. Since Seplite LX120 is an anionic support that has a positive charge, it can interact with negatively charged EstD9 through electrostatic interactions. These interactions can affect the strength of the enzyme attachment and the stability of the immobilized enzyme on the support surface. The other possible explanation of its stabilization mechanism is that the localization of the dehydrated enzyme molecules in parts of the support surface provided mutual spatial fixation that prevent protein unfolding and intermolecular inactivation processes in a water-free state. Therefore, the stability for long storage of immobilized EstD9 compared to the free counterpart verifies its practicability for industrial usages.

The repeated use of the support materials in reaction batches can counter environmental concerns and render the immobilized enzyme more advantageous than its free form [52]. Figure 10b shows the effect of repeated use of the immobilized EstD9 at a reaction temperature of 80 °C and pH 7 to ascertain its recovery when introduced to a new substrate solution. The yield at cycle 0, corresponding to the first reaction, was used as the reference (e.g., 100% conversion), and the conversion of the remaining cycles was calculated accordingly. As depicted in the graph, the immobilized EstD9 exhibited high relative activity after seven consecutive cycles of assay (each 12 h) with only 45.4% of reduction in activity. This indicated that the immobilized EstD9 retained good operational stability. Other research showed that immobilized carboxylesterase EstH could be used for up to 8 cycles, whereas immobilized *L. plantarum* esterase could only retain its activity for three uses [53,54]. The enhanced reusability of immobilized EstD9 may be attributed to electrostatic interaction between the carboxylate group of the amino acid backbone and the support, which preserved enzyme orientation and structural integrity. Therefore, a single batch of immobilized EstD9 production can be used repeatedly and continuously without hindering its functionality. Non-specific adsorption typically has a higher rate of reusability than other techniques for immobilized enzymes [55]. On that account, biotechnological industries are likely to gain benefits from the application of immobilized EstD9.

## 4. Conclusions

The purified EstD9 was successfully immobilized onto a polymer support, Seplite LX120, by physical adsorption, as evaluated with SEM, FT-IR and BET analysis. In reference to infrared spectroscopy, the formation of the functional group was clear evidence of structural modifications of the EstD9 when attached to the surface of the support. In addition, BET quantitative analysis revealed a decrease in specific surface area and total pore volume after immobilization. Under optimal conditions, the immobilized EstD9 exhibited high thermal stability at 10–100 °C, showing that the immobilization method stably maintained structural integrity and stability. The immobilized EstD9 exhibited better tolerance towards various organic solvents and metal ions in comparison to its free state. The improved storage stability, coupled with the repeated use of the enzyme, clearly shows that the enzyme is feasible and economically worthwhile. Accordingly, the strategy of enzyme modification by immobilization satisfies industrial requirements for replacing the conventional practise.

## Data Availability

Not applicable.
